# The forehead and glabella show pronounced *Cutibacterium* relative abundance differences between acne and healthy groups: a regional facial 16S rRNA gene sequencing study

**DOI:** 10.3389/fmicb.2026.1853291

**Published:** 2026-07-15

**Authors:** Wenjing Zha, Ke Li, Yitao Qian, Lechen Zhao, Szeman Cheung, Jianhua Huang, Fei Miao, Jie Shen, Yiwu Yu, Xinran Hu, Hongwei Wang, Ting Lyu, Lei Shi

**Affiliations:** 1 Department of Dermatology, Shanghai Key Laboratory of Clinical Geriatric Medicine, Shanghai Institute of Geriatric Medicine, Huadong Hospital, Fudan University, Shanghai, China; 2 Fudan University, Shanghai, China

**Keywords:** 16S rRNA gene sequencing, acne vulgaris, *Cutibacterium*, facial skin microbiome, regional heterogeneity

## Abstract

**Introduction:**

Acne vulgaris is a prevalent chronic inflammatory skin disease, and Cutibacterium overproliferation is a core pathogenic factor, yet findings remain controversial, partly due to single anatomical area sampling bias.

**Methods:**

We conducted a crosssectional study with anatomically paired sampling across multiple facial anatomical areas in 85 subjects (30 healthy subjects, 55 acne subjects), analyzing 763 samples from 6 facial anatomical areas for 16S rRNA V4 region sequencing and microbial community analysis.

**Results:**

At the whole-face level, acne subjects had a significantly higher relative abundance of Cutibacterium and Staphylococcus compared with healthy subjects. Critically, Cutibacterium enrichment was highly dependent on facial anatomical areas: only the forehead and glabella showed significant intergroup differences, with the forehead showing a 127% higher median relative abundance (adjusted *q* = 0.043) and the glabella showing a 53% higher median relative abundance (adjusted *q* = 0.043), while Staphylococcus enrichment was widespread (present in 5 of 6 areas, except the jaw), and Corynebacterium showed no intergroup differences in any facial area, although LEfSe classified it as a health-associated taxon. Tax4Fun-based functional prediction suggested upregulated antimicrobial resistance and adaptation pathways, and downregulated core metabolic pathways, in acne-associated microbiota.

**Discussion:**

This study demonstrates the facial anatomical area specificity of Cutibacterium enrichment, identifies the forehead and glabella as the areas with pronounced relative abundance differences between acne and healthy groups.

## 1 Introduction

Acne vulgaris is a highly prevalent, chronic inflammatory, and disfiguring skin disease that predominantly affects the face (Reynolds et al., 2024). Its characteristic primary lesions include non-inflammatory comedones, as well as inflammatory papules, pustules, and nodules. Beyond severely impairing patients’ appearance and self-image, the condition is strongly associated with adverse psychological outcomes such as anxiety and depression (Zhu et al., 2025; Chen et al., 2025). To date, the precise pathogenesis of acne vulgaris remains incompletely elucidated (Jin et al., 2023; Podwojniak et al., 2025).

*Cutibacterium acnes* is widely recognized as the key opportunistic pathogen driving cutaneous inflammatory responses in acne (Plackett, 2025; Wang et al., 2018). However, controversy persists regarding whether *Cutibacterium* undergoes “overproliferation” in the skin of acne patients (Podwojniak et al., 2025; Scharschmidt and Segre, 2025; Repac et al., 2025). Fitz-Gibbon et al. (2013) found no statistically significant difference in the relative abundance of *Cutibacterium* between acne and healthy groups via sampling from the nasal tip (Fitz-Gibbon et al., 2013); in contrast, recent studies focusing on the glabellar or forehead areas have reported a significant elevation of *Cutibacterium* relative abundance in acne groups (Witkam et al., 2026; Maître et al., 2024). Collectively, published studies remain inconclusive as to whether the relative abundance of *Cutibacterium acnes* is increased among acne patients.

Multiple factors lead to inconsistent findings across relevant studies. *Cutibacterium acnes* presents obvious strain-level heterogeneity. Different phylotypes, such as phylotype IA1 linked to acne and phylotype II associated with healthy skin, vary in virulence and pro-inflammatory capacity, which is an intrinsic factor contributing to divergent research conclusions (Dagnelie et al., 2018; Scholz et al., 2014). In addition, *Staphylococcus* species (e.g., *Staphylococcus epidermidis*) have also been suggested to participate in skin microbiota dysbiosis during acne development (Hamann et al., 2025). However, their pathogenic potential, regional distribution characteristics on the face and intergroup differences remain unclear. Furthermore, most prior studies have adopted a single facial anatomical area sampling strategy, which may overlook the inherent microenvironmental heterogeneity across different facial areas in terms of sebaceous gland density, skin hydration, and skin dryness (Conwill et al., 2022). Anatomical area-related sampling bias may partially explain the discrepancies in these previous results. Few studies to date have employed multi-facial anatomical area anatomically paired sampling between acne and healthy subjects to explore the role of the skin microbiota in acne pathogenesis (Jin et al., 2023).

A large proportion of studies on facial microbiota adopt 16S rRNA gene sequencing for genus-level analyses. To date, the distribution patterns of facial microbiota in acne patients have not been systematically investigated based on a standardized paired multi-area sampling design. This study focused on genus-level microbial community structure and facial regional distribution profiles, and explored the regional specificity of microbial differences. Although 16S rRNA gene sequencing cannot resolve strain-level variations and has inherent limitations in taxonomic resolution, identifying the differentially enriched facial areas of acne-associated core genera can provide a preliminary basis for subsequent high-resolution strain research and the development of standardized microbiota sampling protocols.

We designed an observational study based on multi- facial anatomical area paired facial sampling. We divided the subjects’ faces into 6 major anatomical areas (forehead, cheek, jaw, glabella, nasal dorsum, and chin), and further subdivided the forehead, cheek, and jaw into left and right subareas. For each area, we performed separate lesion status assessment (healthy area, non-inflammatory lesion area, or inflammatory lesion area) and skin swab sampling, followed by sequencing of the V4 hypervariable region of the 16S rRNA gene. By integrating data from a total of 763 samples, this study aims to systematically characterize the spatial heterogeneity of the facial microbial community, with a focus on investigating the regulatory effects and potential interactions of facial anatomical area and acne disease status on the relative abundance of *Cutibacterium*. We preliminarily identified area-dependent enrichment differences among key acne-associated genera. These results help explain existing study controversies from a microecological perspective. The forehead and glabella showed marked intergroup disparities, which may guide sampling site choices in future acne microbiome studies.

## 2 Materials and methods

### 2.1 Study design

This study was designed as a cross-sectional study. The target population of this study comprised individuals with facial acne vulgaris and healthy individuals without facial acne lesions. Subjects were enrolled from the Dermatology Outpatient Clinic of Huadong Hospital Affiliated to Fudan University, or via poster recruitment, between August 25, 2025 and October 25, 2025. This study protocol was approved by the Ethics Committee of Huadong Hospital Affiliated to Fudan University (Approval No.: 2025 K300), and written informed consent was obtained from all subjects or their legal guardians. This study was conducted in strict accordance with the Declaration of Helsinki.

### 2.2 Inclusion and exclusion criteria for subjects

#### 2.2.1 Inclusion criteria

Aged between 8 and 40 years; voluntarily participated in this clinical study, signed the informed consent form, and were able to cooperate with clinical examinations. For acne subjects: facial lesions met the clinical diagnostic criteria for acne vulgaris in the 2024 Edition of the Guidelines for the Management of Acne Vulgaris issued by the American Academy of Dermatology (Reynolds et al., 2024). For healthy subjects: no acne vulgaris lesions on the face.

#### 2.2.2 Exclusion criteria

Received oral immunosuppressants, glucocorticoids, or contraceptives within the past 3 months; received oral medications including antibiotics or isotretinoin within the past 2 months; received facial photoelectric therapy or topical drug treatment within the past 2 weeks; had other concurrent facial skin diseases at enrollment, including rosacea, acne induced by topical oils or ointments, drug-induced acneiform eruptions, and seborrheic dermatitis; had a history of facial trauma, skin grafting, or facial surgery; had severe systemic diseases; were pregnant or breastfeeding.

### 2.3 Area division and area lesion status assessment criteria

This study referred to relevant facial anatomical studies (Nguyen and Duong, 2026) and divided the subjects’ faces into 6 core anatomical areas: forehead, cheek, jaw, glabella, nasal dorsum, and chin. The forehead, cheek, and jaw are bilaterally symmetric and were further divided into left and right subregions. The glabella, nasal dorsum, and chin are midline areas and were sampled as single sites. Thus, a total of nine sampling sites were collected per subject: left forehead, right forehead, left cheek, right cheek, left jaw, right jaw, glabella, nasal dorsum, and chin. The lesion status of each area was assessed clinically by one senior dermatologist, and the assessment results were determined based on the morphological characteristics of skin lesions in each facial area.

For acne subjects, according to the clinical manifestations of skin lesions, each facial anatomical area was categorized into one of the following three types (Force, 2019), based on which the acne subjects were further divided into subgroup:

Healthy Area (HA): No visible acne vulgaris lesions of any type are present in this area;

Non-Inflammatory Lesion Area (NIA): The area is dominated by open comedones and/or closed comedones, with no inflammatory lesions (papules, pustules, nodules, or cysts) present;

Inflammatory Lesion Area (IA): Any inflammatory lesions (papules, pustules, nodules, or cysts) are observed, regardless of the presence of comedones.

### 2.4 Demographic information

The following host variables were collected by the researchers: gender, age, body mass index (BMI), and the time interval since the last face cleansing before sampling.

### 2.5 Sample collection

To capture the in-situ facial microbiota under real-world conditions, participants followed their habitual face-washing, skincare, and makeup routines prior to sampling, without any additional disinfection, cleansing, or makeup removal. All samples were collected by a single dermatologist following a standardized protocol. A flocked swab (Genecome, Cat. No. YJY-ZR-15CM) was moistened in sterile 0.9% normal saline solution, then firmly rubbed against the target area (approximately 4 cm^2^) with 40 sequential wipes to ensure adequate microbial collection. Care was taken to avoid contact with non-target surfaces.

After sampling, the swab handle was broken at the break mark, and the swab tip was placed into a sterile cryopreservation tube (Servicebio, Cat. No. CV-3). The tube was snap-frozen in liquid nitrogen within 20 min, and stored at −80 °C for up to 1 week and transported on dry ice within 48 h of collection, and subsequent 16S rRNA gene sequencing was entrusted to Novogene Co., Ltd. (Beijing, China).

### 2.6 Library construction and high-throughput sequencing

All experimental procedures for genomic DNA extraction, 16S rRNA gene library construction, and high-throughput sequencing were performed by Novogene Co., Ltd. (Beijing, China) following their standardized and validated commercial workflows.

The V4 hypervariable region of the 16S rRNA gene was amplified using the primer pair 5’-GTGCCAGCMGCCGCGGTAA-3′ and 5’-GGACTACHVGGGTWTCTAAT-3′. The qualified PCR amplicons were verified via capillary electrophoresis (Agilent 5,400 Automated Capillary Electrophoresis System), then pooled, purified, and used for library preparation. The library was constructed via end repair, A-tailing, sequencing adapter ligation, and purification, then quantified using a Qubit fluorometer and real-time quantitative PCR. Upon library qualification, paired-end 250 bp (PE250) sequencing was performed on the Illumina NovaSeq 6000 platform.

### 2.7 Statistical methods

Raw sequencing reads were subjected to quality filtering using Trimmomatic. Bases with Phred quality scores below 20 at both ends were trimmed. A sliding window approach was applied with a window size of 4 bp and a mean quality threshold of 15. Only sequences longer than or equal to 100 bp were retained. The qualified reads were merged with FLASH. The DADA2 algorithm was used to identify Amplicon Sequence Variants (ASVs) and remove chimeric sequences. Two types of negative controls were set in this study, including enzyme-free ultrapure water and kit blank controls. All ASVs detected in negative controls were excluded. Rarefaction was performed on the ASV abundance table to normalize sequencing depth. Singleton ASVs (those with a total read count of 1 across all samples) were further filtered out. Finally, taxonomic annotation was implemented on the QIIME2 platform using the SILVA 138 reference database.

The Shannon index was calculated to reflect the overall community alpha diversity. Linear Mixed Models (LMMs) built via the R package lme4 were used to adjust for repeated measurements from multiple sampling sites of the same subject. In the model, grouping was defined as the fixed effect and subject ID as the random intercept. Parameter estimation was performed using Restricted Maximum Likelihood (REML). The Satterthwaite method was adopted to compute degrees of freedom, and pairwise comparisons between groups were conducted with the Kenward-Roger method. Given that each subject had multiple samples collected from different facial sites, ASV abundances were aggregated by subject using the median value to eliminate pseudoreplication caused by repeated measures. A total of 85 independent observational samples were obtained. Based on the Bray-Curtis distance matrix, Permutational Multivariate Analysis of Variance (PERMANOVA) was carried out with 999 permutations. Meanwhile, Permutational Multivariate Dispersion (PERMDISP) was used to compare differences in community dispersion across groups.

The Linear Discriminant Analysis Effect Size (LEfSe) algorithm was used to screen differentially abundant taxa between groups with default parameters. The threshold of Linear Discriminant Analysis (LDA) was set to 4.0, and the significance level of the Kruskal-Wallis test was *α* < 0.05. Since LEfSe cannot correct for within-subject repeated measures, no corresponding adjustment was applied in this analysis.

Microbial functional potential was predicted using Tax4Fun against the KEGG database, based on taxonomic classification results of 16S rRNA gene sequences. Linear Mixed Models with the formula Functional pathway abundance ~ Group + (1 | Subject ID) were constructed for statistical analysis of repeated sampling data. Raw *p*-values of all pathways were corrected for multiple testing using the Benjamini-Hochberg procedure to control the False Discovery Rate (FDR). Differences with a corrected FDR < 0.05 were considered statistically significant.

Generalized Estimating Equations (GEE) were applied to compare genus-level abundances across the whole face, adjusting for within-subject repeated measures. GEE combined with Sequential Bonferroni multiple testing correction was also used for comparisons between different lesion subgroups and the healthy group.

Chi-squared test combined with standardized residual analysis was used to explore the association between facial anatomical regions and local lesion status, including HA, NIA and IA. A heatmap of standardized residuals was plotted for visualization. A |standardized residual| > 1.96 was taken as the cutoff for statistically significant differences in community composition between groups.

For facial regional specificity analysis: abundances from left and right subregions of bilaterally symmetrical facial areas (forehead, cheeks, jaw, etc.) were averaged per subject, generating six independent datasets corresponding to different facial regions. The Mann–Whitney U test was performed for each region separately, and the Benjamini-Krieger-Yekutieli (BKY) method was used for multiple testing correction with a preset FDR controlled below 10%. A corrected q-value < 0.05 was defined as statistically significant.

## 3 Results

### 3.1 Demographic characteristics of subjects

**Figure 1:**
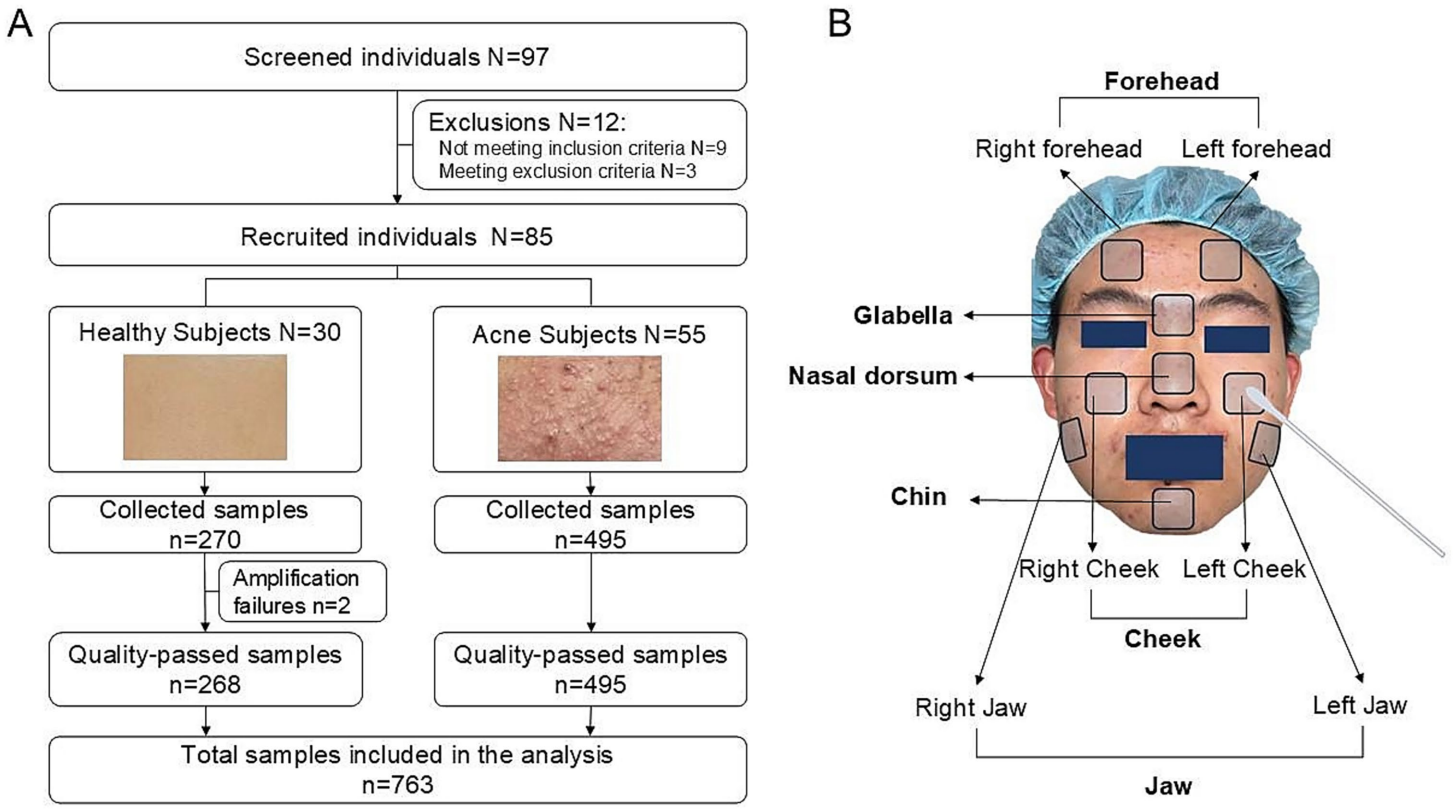
Study flow chart and standardized facial sampling protocol. **(A)** Flow chart of subject enrollment, sample collection and quality control. A total of 97 individuals were screened, 12 were excluded (9 did not meet the inclusion criteria, 3 met the exclusion criteria), and 85 subjects (30 healthy subjects and 55 acne subjects) were finally enrolled. A total of 765 samples were collected, and 763 valid samples were included in the subsequent 16S rRNA gene sequencing analysis after strict quality control. **(B)** Schematic diagram of the standardized facial sampling scheme. A total of 9 swabs were collected per subject, covering 6 major facial anatomical areas: forehead, cheek, jaw, glabella, nasal dorsum, and chin. The forehead, cheek, and jaw were sampled bilaterally, while the glabella, nasal dorsum, and chin were sampled at the midline.

A total of 85 subjects were finally enrolled in this study, including 30 healthy subjects and 55 acne subjects (Figure 1A). Demographic baseline characteristics including age, gender and body mass index (BMI) were well balanced and comparable between the two groups (all *p* > 0.05). Detailed demographic information and relevant lifestyle data are presented in Table 1.

**Table 1:** Demographic information of subjects.

**Variable**	**Overall** *N* = 85	**HS** *N* = 30	**AS** *N* = 55	**Test statistic**	***p*-value**
Gender				χ^2^ = 0.20	0.66
Female, *N* (%)	54 (63.53%)	20 (66.67%)	34 (61.82%)		
Male, *N* (%)	31 (36.47%)	10 (33.33%)	21 (38.18%)		
Age, years, Median (IQR)	23.00 (19.00–26.00)	23.00 (19.00–25.20)	22.00 (19.00–27.00)	U = 799.50	0.82
BMI, kg/m^2^, Median (IQR)	21.26 (18.67–23.35)	21.48 (19.40–23.10)	21.05 (18.25–23.57)	U = 758.50	0.54
Time interval since the last face cleansing before sampling				Fisher’s exact test	0.82
≤1 h, *N* (%)	5 (5.88%)	1 (3.33%)	4 (7.27%)		
1–3 h, *N* (%)	24 (28.24%)	10 (33.33%)	14 (25.45%)		
3–6 h, *N* (%)	22 (25.88%)	7 (23.33%)	15 (27.27%)		
≥6 h, *N* (%)	34 (40.00%)	12 (40.00%)	22 (40.00%)		

Using a standardized facial sampling protocol (9 swabs were collected per subject, covering 6 major facial anatomical areas: forehead, cheek, jaw, glabella, nasal dorsum, and chin. The forehead, cheek, and jaw were sampled bilaterally, while the glabella, nasal dorsum, and chin were sampled at the midline; Figure 1B), a total of 763 valid samples were obtained for 16S rRNA gene sequencing targeting the V4 hypervariable region after rigorous quality control (Figure 1A). Library construction and sequencing were successfully completed for all included samples.

### 3.2 Altered facial microbial diversity and dominant bacterial genera composition in acne subjects

**Figure 2:**
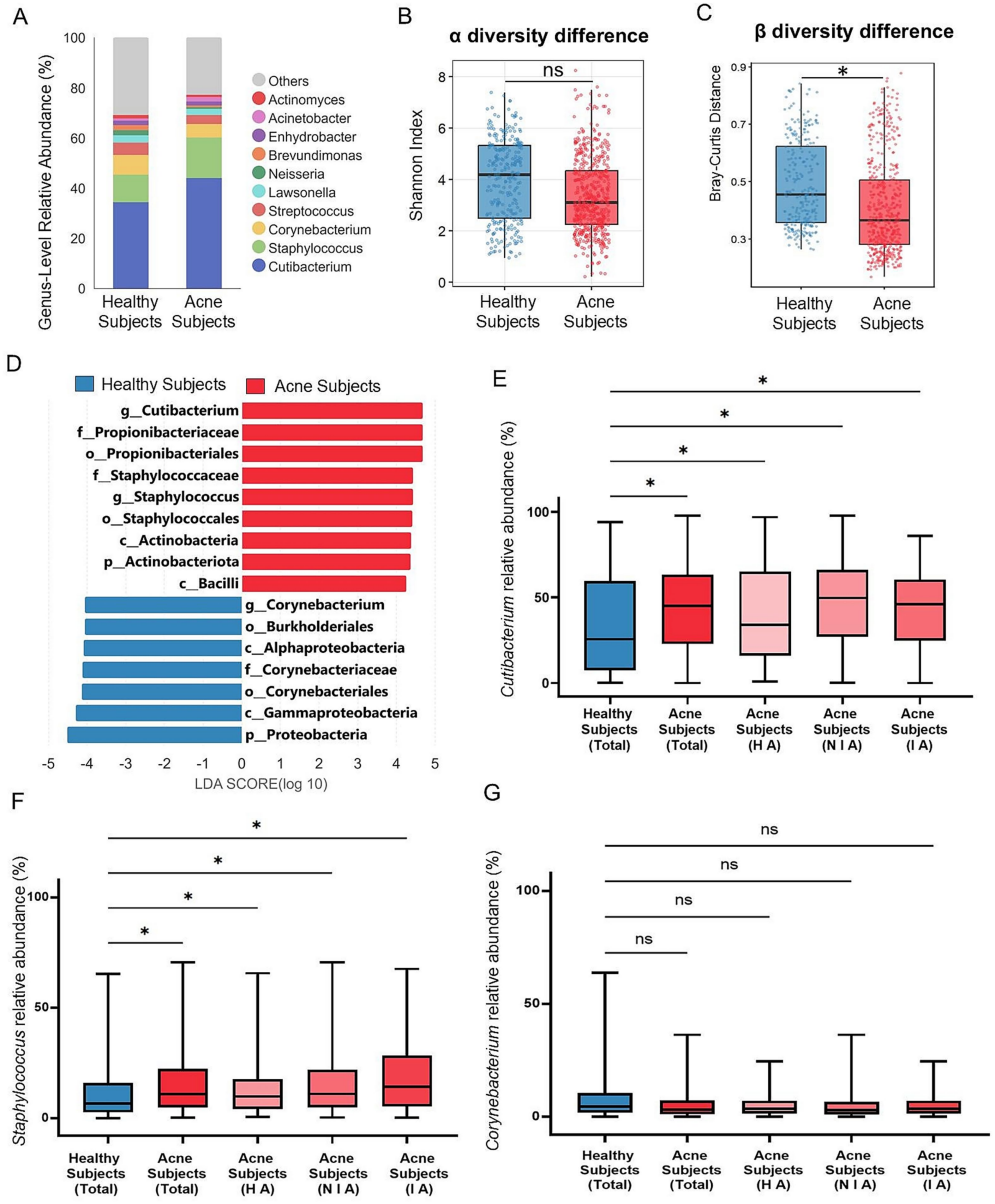
Differences in facial skin microbiota composition, diversity, and dominant bacterial genera relative abundance between healthy subjects and acne subjects. **(A)** Stacked bar chart showing genus-level relative abundance of the dominant skin microbiota in healthy subjects and acne subjects at the whole-face level. Taxa with low abundance were merged into others. **(B)** Box plot comparing *α*-diversity (Shannon index) of facial skin microbiota between healthy subjects and acne subjects. Statistical method: Linear Mixed Models (LMMs) were used to assess intergroup differences, with subject ID treated as a random effect to adjust for repeated sampling from the same subject. The Kenward-Roger method was used for pairwise comparisons, with statistical significance set at *p* < 0.05. **(C)** Box plot comparing *β*-diversity (Bray–Curtis dissimilarity) of facial skin microbiota between healthy subjects and acne subjects. Statistical method: ASV abundances were aggregated by subject median to eliminate pseudoreplication. Permutational Multivariate Analysis of Variance (PERMANOVA) with 999 permutations was performed to assess intergroup differences in community composition, and a permutation test for homogeneity of dispersions was used to assess differences in within-group variability. Statistical significance was set at *p* < 0.05. **(D)** LEfSe analysis identifying differentially abundant microbial biomarkers between the two groups. Statistical method: Kruskal-Wallis test was used for significance assessment (α < 0.05), with an LDA score threshold of > 4.0 for biomarker selection. No repeated measure correction was performed in this analysis. **(E)** Box plot comparing the relative abundance of *Cutibacterium* among healthy subjects, total acne subjects, and acne subgroups stratified by lesion status (HA, NIA, IA). **(F)** Box plot comparing the relative abundance of Staphylococcus among healthy subjects, total acne subjects, and acne subgroups stratified by lesion status (HA, NIA, IA). **(G)** Box plot comparing the relative abundance of *Corynebacterium* among healthy subjects, total acne subjects, and acne subgroups stratified by lesion status (HA, NIA, IA). General statistical method for **(E–G)**: Generalized Estimating Equations (GEE) were used to assess intergroup differences, with subject ID treated as a random effect to adjust for repeated sampling from the same subject. Bonferroni correction was applied for multiple pairwise comparisons, with statistical significance set at *p* < 0.05.

At the whole-face level, the facial microbial community structure differed between acne subjects and healthy subjects. Genus-level analysis showed that *Cutibacterium* was the dominant genus in both groups (Figure 2A). For microbial diversity, acne subjects exhibited a trend of lower *α*-diversity (Shannon index) compared with healthy subjects, though the difference did not reach statistical significance (*p* = 0.052) (Figure 2B). In contrast, *β*-diversity (Bray–Curtis distance) was significantly lower in acne subjects, with both within-group dispersion and overall community composition showing significant intergroup differences (*p* < 0.05) (Figure 2C).

LEfSe analysis was performed to screen for differential microbial biomarkers between groups, and the results showed that *Cutibacterium* and *Staphylococcus* were acne-associated microbial biomarkers, while *Corynebacterium* was enriched in the healthy subjects (LDA > 4.0; Figure 2D). Further analysis by GEE indicated that acne subjects had significantly higher relative abundance of *Cutibacterium* and *Staphylococcus* at the whole-face level (both *p* < 0.05), while no significant intergroup difference was observed for *Corynebacterium* (*p* > 0.05). Stratified analysis by local lesion status (HA, NIA, IA) showed that: *Cutibacterium* relative abundance was significantly elevated in all three acne subgroups (HA, NIA, IA) compared with healthy subjects, with the most pronounced increase observed in the NIA subgroup; *Staphylococcus* relative abundance was significantly higher in all acne subgroups relative to healthy controls; no statistically significant differences in *Corynebacterium* relative abundance were observed between any acne subgroup and healthy subjects (Figures 2E–G).

### 3.3 The difference in *Cutibacterium* relative abundance across subjects and lesional statuses was anatomical area-dependent

**Figure 3:**
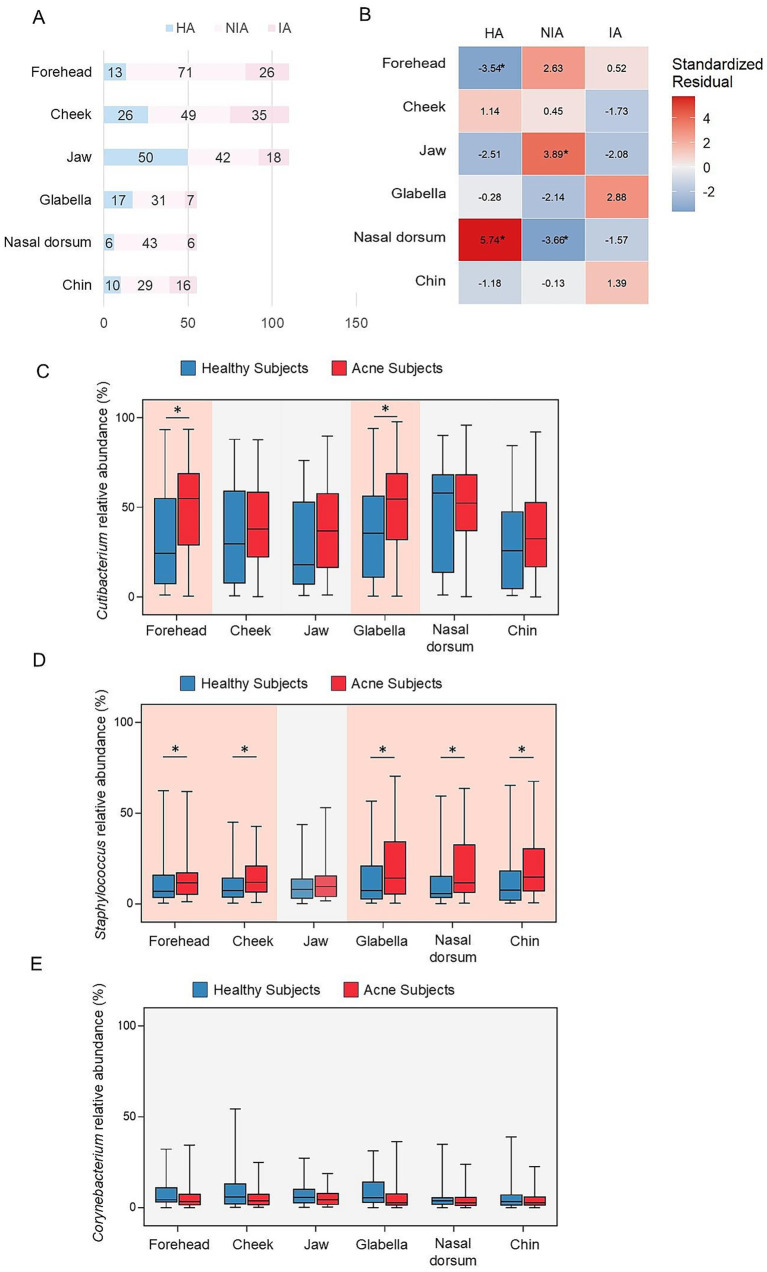
Distribution of acne lesions across facial anatomical areas, association between facial anatomical areas and local lesion status, and area-specific relative abundance of *Cutibacterium*, *Staphylococcus*, and *Corynebacterium*. **(A)** Bar chart showing the distribution of the number of HA, NIA, and IA across 6 major facial anatomical areas (forehead, cheek, jaw, glabella, nasal dorsum, chin) in acne subjects. **(B)** Heatmap of standardized residuals showing the association between facial anatomical areas and local lesion status. Statistical method: Chi-squared test was first performed to assess the overall association between facial anatomical regions and local lesion status (HA, NIA, IA), with the result showing a statistically significant overall association (*p* < 0.05). Subsequently, standardized residual analysis was conducted based on the Chi-squared test result to calculate the standardized residual for each combination of facial anatomical area and lesion status. A |standardized residual| > 1.96 was taken as the cutoff for statistically significant differences, with corresponding *p*-values calculated for each combination. The heatmap of standardized residuals was plotted for visualization of the specific association patterns. *p < 0.05. **(C)** Box plots comparing the relative abundance of *Cutibacterium* in each of the 6 facial anatomical areas between healthy subjects and acne subjects. **(D)** Box plots comparing the relative abundance of *Staphylococcus* in each of the 6 facial anatomical areas between healthy subjects and acne subjects. **(E)** Box plots comparing the relative abundance of *Corynebacterium* in each of the 6 facial anatomical areas between healthy subjects and acne subjects. General statistical method for **(C–E)**: the relative abundance of bilateral symmetric facial areas (forehead, cheek, jaw) was averaged per subject to avoid pseudoreplication from repeated sampling of the same subject. Intergroup comparisons for each facial area were performed using the Mann–Whitney *U* test, with the BKY procedure applied for FDR correction to obtain adjusted *q* values. Statistical significance was set at *q* < 0.05.

Comparative analysis of the left and right hemifaces in healthy controls revealed a high symmetry in microbiota composition and diversity between bilateral paired areas, while considerable heterogeneity in basal microbial community structure was observed among the 6 core facial anatomical areas. In acne subjects, the local lesion status of the left and right hemifaces was also relatively symmetrical, while the distribution of local lesion status (HA, NIA, IA) varied across the 6 anatomical areas (Figure 3A).

To investigate the distribution characteristics of acne lesions across different facial anatomical areas, we first performed Chi-squared test to evaluate the overall association between facial anatomical areas and local lesion status (HA, NIA, IA). The significant results were as follows: The forehead showed a significant negative association with HA (standardized residual = −3.540, *p* = 0.0072); The nasal dorsum showed a significant positive association with NIA (standardized residual = 3.887, *p* = 0.0018); The jaw showed a significant positive association with HA (standardized residual = 5.742, *p* < 0.0001) and a significant negative association with NIA (standardized residual = −3.661, *p* = 0.0045); No other statistically significant associations were observed between the remaining facial anatomical areas and lesion statuses. The specific association patterns were visualized via a heatmap of standardized residuals (Figure 3B).

Given the significant heterogeneity in basal microbiota structure and local lesion distribution across different facial areas, we further investigated whether anatomical area affects the difference in *Cutibacterium* relative abundance between acne subjects and healthy subjects. We performed area-matched intergroup comparisons, with the relative abundance of bilateral symmetric areas (forehead, cheek, jaw) averaged per subject to avoid pseudoreplication, and intergroup differences assessed via the Mann–Whitney U test with BKY FDR correction for 6 multiple comparisons, consistent with our pre-specified statistical analysis plan. The results showed that only the forehead and glabella areas had significant intergroup differences after FDR correction, while no significant differences were observed in the cheek, jaw, nasal dorsum and chin areas. The increase in the forehead area was the most prominent: the median relative abundance of *Cutibacterium* rose from 24.17% in healthy controls to 54.89% in acne patients, a 127% relative elevation with a statistically significant difference (adjusted *q* = 0.043). Notably, the nasal dorsum area of healthy controls exhibited an extremely high basal relative abundance of *Cutibacterium* (median 57.89%), and no significant change in its abundance was observed in acne subjects (median 52.35%, adjusted *q* = 0.72) (Figure 3C). In contrast to the area-specific enrichment of *Cutibacterium*, *Staphylococcus* showed widespread significant elevation in acne patients across almost all facial anatomical areas, including the forehead, cheek, glabella, nasal dorsum and chin (all adjusted *q* < 0.05), with only the jaw area showing no significant intergroup difference (adjusted *q* > 0.05) (Figure 3D). For *Corynebacterium*, although whole-face level analysis showed a slight overall reduction in acne patients, no statistically significant intergroup differences were observed in any of the 6 facial anatomical areas after BKY-FDR correction (all adjusted q > 0.05), with its relative abundance remaining stable across all areas regardless of acne disease status (Figure 3E).

### 3.4 Functional predictions associated with acne vulgaris

**Figure 4:**
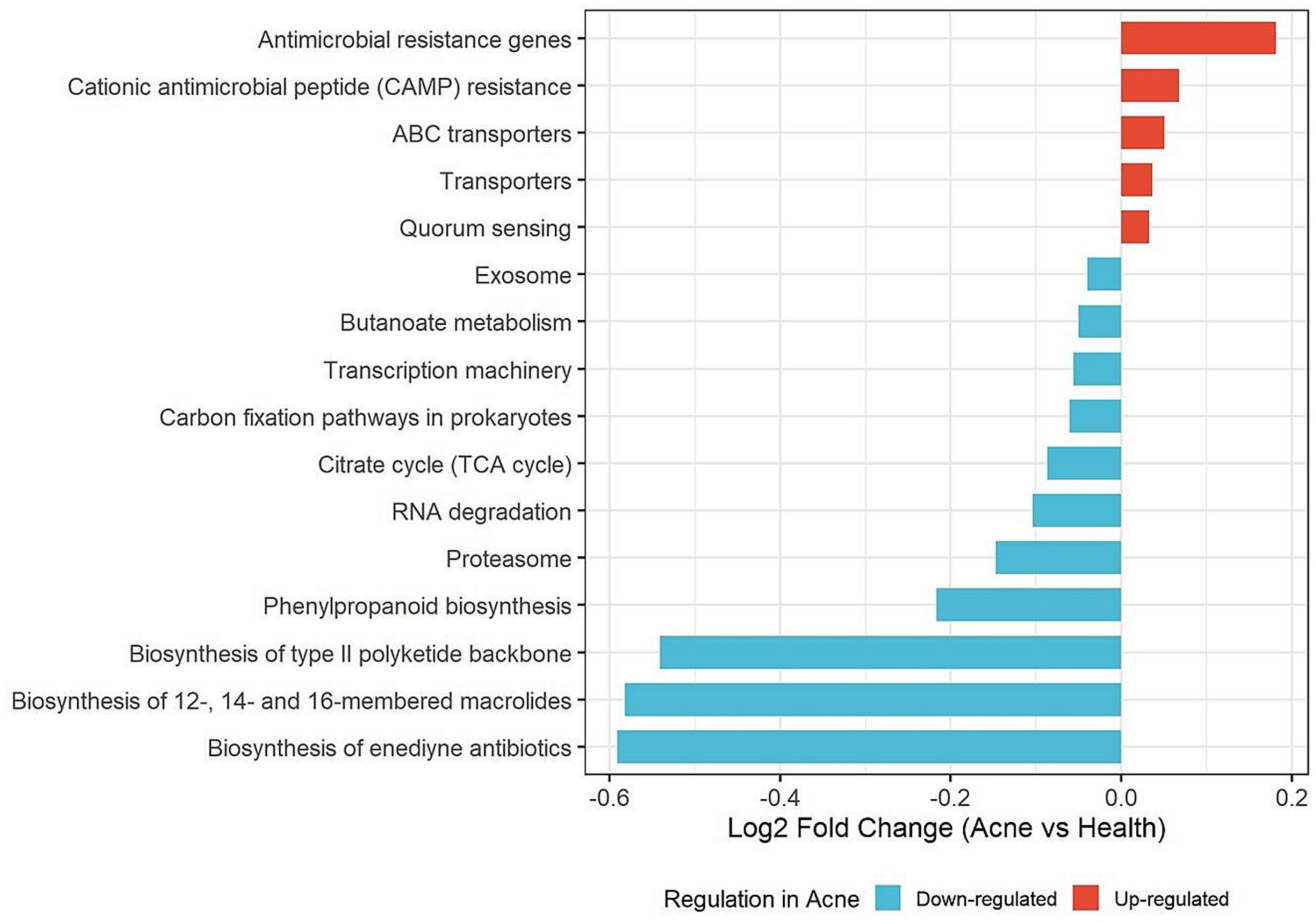
Fold change of acne-associated differential functional pathways in facial skin microbiota. Microbial functional potential was predicted via Tax4Fun against the KEGG database based on 16S rRNA gene sequencing results, with all results being predicted values rather than experimentally measured activities. Significantly differential pathways (corrected FDR < 0.05) were identified via Linear Mixed Models and multiple testing correction, and the Log2 Fold Change of pathways in the acne group relative to healthy controls was visualized, with red for upregulated pathways and blue for downregulated pathways.

To investigate the functional perturbation characteristics of facial skin microbiota during the pathogenesis of acne vulgaris, this study performed prediction of microbial functional potential using the Tax4Fun tool against the Kyoto Encyclopedia of Genes and Genomes (KEGG) database, based on the taxonomic classification results of 16S rRNA gene sequencing. It should be explicitly noted that all functional results reported in this section are predicted values derived from the 16S rRNA gene taxonomic profile, rather than experimentally measured functional activities. Intergroup difference analysis was conducted for the repeated sampling data, and raw *p*-values of all pathways were corrected via multiple testing correction. Differences with a corrected FDR < 0.05 were considered statistically significant. This study focused on all significantly differential functional pathways, and compared the predicted microbial functional profiles between acne subjects and healthy controls (Figure 4).

A total of 16 significantly differential functional pathways were identified between the two groups. Among them, 5 pathways were significantly enriched in the acne vulgaris group, which were mainly related to bacterial environmental adaptation, stress resistance, and population behavior regulation. The core upregulated pathways included Antimicrobial resistance genes, Cationic antimicrobial peptide (CAMP) resistance, ABC transporters, Transporters, and Quorum sensing. Specifically, ABC transporters and Transporters are the core functional modules for bacterial nutrient uptake, efflux of toxic substances, and resistance to external environmental stress; Antimicrobial resistance genes and CAMP resistance pathway are closely associated with the ability of bacteria to evade host immune defense and resist antimicrobial interventions; Quorum sensing pathway is responsible for coordinating bacterial population behavior and regulating the expression of virulence-related genes. The predicted upregulation of these pathways offer hypothesis-generating clues for the over-proliferation and enhanced environmental adaptability of acne-associated pathogenic bacteria in facial skin.

In contrast, the remaining 11 significantly differential pathways were significantly enriched in the healthy control group, which were mainly involved in bacterial basal metabolism, biosynthesis of antimicrobial compounds, and maintenance of cellular homeostasis. The core pathways included Biosynthesis of enediyne antibiotics, Biosynthesis of 12-, 14- and 16-membered macrolides, Biosynthesis of type II polyketide backbone, Phenylpropanoid biosynthesis, Proteasome, RNA degradation, Citrate cycle (TCA cycle), Carbon fixation pathways in prokaryotes, Transcription machinery, Butanoate metabolism, and Exosome. Among them, the biosynthesis pathways of antibiotics and polyketides are related to the production of antimicrobial compounds by commensal bacteria, which contribute to the inhibition of pathogenic bacterial over-proliferation; basal metabolic pathways such as TCA cycle, carbon fixation, and transcription machinery are the core for maintaining the normal physiological functions and metabolic homeostasis of commensal bacteria; Proteasome and RNA degradation pathways are involved in the regulation of cellular protein and RNA turnover, which are essential for maintaining bacterial cellular homeostasis. The predicted enrichment of these pathways in the healthy control group imply a possible contribution to skin microecological homeostasis of healthy facial skin microecology.

## 4 Discussion

This study performed observational analysis using paired sampling based on facial anatomical areas, providing new insights to clarify the controversies surrounding alterations in the abundance of *Cutibacterium acnes* (Schneider et al., 2023). First, we compared the overall facial microbiota and microbial diversity between the acne group and the healthy control group, and detected distinct differences in microbial composition and diversity across the two cohorts. Subsequently, LEfSe analysis was applied to identify signature genera for each group. The results showed that *Cutibacterium* and *Staphylococcus* were the signature genera of the acne subjects, while *Corynebacterium* dominated in the healthy subjects. All samples from the healthy subjects were set as controls for further comparisons with the overall acne group and its lesion subgroups, namely HA, NIA and IA. We then analyzed changes in the relative abundance of *Cutibacterium*, *Staphylococcus* and *Corynebacterium*. At both the overall and subgroup levels, the relative abundances of *Cutibacterium* and *Staphylococcus* were significantly higher in acne subjects than in healthy subjects, whereas the relative abundance of *Corynebacterium* was lower in the acne subjects at the overall level.

We further conducted area-matched comparisons between the two groups. The microbial community structure exhibited a symmetric distribution in bilateral facial subregions (left and right forehead, cheeks and jaw). Given this symmetric feature, we calculated the mean abundance of each taxon for paired bilateral subregions prior to subsequent analyses. The intergroup differences in *Cutibacterium* relative abundance presented area-dependent patterns, with statistically significant differences only observed in the forehead and glabella. The most pronounced difference was found in the forehead: the median relative abundance in acne patients was 127% higher than that in healthy controls (adjusted *q* = 0.043). No significant intergroup difference was detected on the nasal dorsum, which may be attributed to the inherently high baseline relative abundance (57.9%) of *Cutibacterium* in healthy individuals at this area. For *Staphylococcus*, significant intergroup differences were identified in five out of six facial areas, excluding the jaw. By contrast, no significant intergroup differences in *Corynebacterium* relative abundance were observed across all tested regions.

Notably, this study characterized the abundance patterns of the three genera at the genus level, which lays a foundation for further in-depth microbiota research. Nevertheless, 16S rRNA gene sequencing has limited taxonomic resolution and cannot distinguish functional variations among different species within the same genus. In fact, substantial inter-species heterogeneity exists among *Cutibacterium*, *Staphylococcus* and *Corynebacterium*. The genus *Cutibacterium* mainly include*s Cutibacterium acnes*, *Cutibacterium granulosum* and *Cutibacterium avidum*, with *Cutibacterium acnes* acting as the absolutely dominant species (Barnard et al., 2016; Byrd et al., 2018; Yu et al., 2024). Accumulating evidence indicates that subspecies/phylotypes of *Cutibacterium acnes* differ in pathogenicity: phylotype IA1 is closely associated with acne, while phylotype II is predominantly detected on healthy skin (Podbielski et al., 2024; Salar-Vidal et al., 2021). *Cutibacterium granulosum* carries virulence and drug resistance genes, and its multidrug-resistant plasmids can be horizontally transferred to *Cutibacterium acnes*, conferring resistance to macrolides and clindamycin; this species is therefore tightly linked to acne pathogenesis (Chen et al., 2024). As an opportunistic pathogen, *Cutibacterium avidum* mainly colonizes moist body sites such as the axilla. Its direct role in facial acne is rarely reported, but it may modulate local microecology via interbacterial interactions (Jiang et al., 2022; Corvec, 2018). Different species within the genus *Staphylococcus* exert divergent effects on acne development. Previous research reported that *Staphylococcus epidermidis* (44.2%) predominated in non-lesional forehead skin of acne patients, whereas *Staphylococcus capitis* (46.7%) was the dominant species in healthy controls (Maître et al., 2024). Distinct lineages of *Staphylococcus epidermidis* possess different biological functions: lineage 1 secretes succinic acid to exert anti-inflammatory effects, while lineage 3 promotes inflammation by forming biofilms and activating the TLR-2 signaling pathway (Hamann et al., 2025). *Staphylococcus aureus* is more frequently detected in acne lesions. It is considered an opportunistic colonizer that proliferates when the skin barrier is impaired, yet its independent pathogenic role in acne remains to be validated (Legiawati et al., 2023). *Corynebacterium* was identified as a signature genus of the healthy group in the present study, and its overall relative abundance was lower in acne subjects. Multiple studies have suggested that *Corynebacterium* species are associated with healthy skin homeostasis. For instance, *Corynebacterium striatum* can attenuate the virulence of *Staphylococcus aureus* and drive it toward a commensal state (Ramsey et al., 2016). *Corynebacterium amycolatum* participates in microbial nutritional interactions by producing cobalamin and plays a vital role in maintaining the stability of healthy skin microbiota (Swaney et al., 2025). *Corynebacterium tuberculostearicum* is a dominant commensal species at multiple skin sites in healthy humans (Ahmed et al., 2023). The specific functions of different *Corynebacterium* species still require further exploration. Significant intergroup differences in *Cutibacterium* relative abundance were only found in the forehead and glabella, but not on the nasal dorsum, and the underlying mechanism remains unclear. One plausible explanation lies in the varied baseline relative abundance of *Cutibacterium* across different facial regions in healthy individuals: the nasal dorsum had the highest median baseline abundance (57.9%), followed by the glabella (35.6%) and forehead (24.2%), while the jaw showed the lowest level (17.97%). The lack of intergroup difference on the nasal dorsum may result from its high baseline abundance and strong ecological stability, leaving limited room for further elevation during disease progression. No significant differences were found in the cheeks, jaw and chin, which may be caused by large intra-group variation among healthy individuals or insufficient sample size for these regions. In addition, 16S rRNA sequencing fails to capture subtle strain-level shifts, such as an increase in pathogenic phylotypes and a reduction in commensal phylotypes on the nasal dorsum and other areas. All the above factors may collectively lead to the area-dependent distribution of intergroup differences. The distribution patterns of *Staphylococcus* and *Corynebacterium* differed markedly from that of *Cutibacterium*. Significant intergroup differences in *Staphylococcus* were present in five of the six facial areas (except the jaw), indicating that alterations of this genus are widespread across the face. The absence of differences in the jaw may be related to its relatively high baseline relative abundance of *Staphylococcus* (7.90%) in healthy people. For *Corynebacterium*, no significant intergroup differences were observed in any of the six anatomical areas.

Functional prediction based on 16S rRNA data using Tax4Fun revealed that compared with healthy controls, the acne-associated microbiota exhibited upregulated pathways related to transporter proteins, ABC transporters, two-component systems and quorum sensing, while pathways involved in basic metabolism such as oxidative phosphorylation were downregulated. These predicted pathways may correlate with bacterial colonization, biofilm formation and quorum sensing, consistent with known acne dysbiosis features. Similar functional profiles have been documented in previous skin microbiome studies (Yu et al., 2024; Cavallo et al., 2022). It should be emphasized that these results are bioinformatic inferences rather than direct measurements of gene expression or metabolic activity, and should be regarded as exploratory hypotheses (Aßhauer et al., 2015).

Several limitations of this study need to be acknowledged. 16S rRNA sequencing only provides relative microbial abundance, lacking the capacity for absolute quantification and differentiation of Cutibacterium acnes phylotypes. Strain-level heterogeneity contributes substantially to acne pathogenesis, and genus-level analysis cannot capture strain-specific changes. A cross-sectional design cannot confirm causal associations. LEfSe differential abundance analysis was not adjusted for repeated measures, and its results are exploratory. Facial anatomical areas, lesion types, sebum secretion activity and acne severity are highly correlated and may cause confounding bias. Regional sebum secretion was not measured, and the sample size was insufficient for confounding adjustment. Uncontrolled daily skincare and makeup habits may also introduce confounders. Tax4Fun functional predictions are theoretical and require validation with metagenomics and metatranscriptomics. Small sample sizes in some subgroups and the single-center nature of this cohort mean our findings need verification in independent populations.

Enrichment of facial *Cutibacterium* in acne patients displays distinct regional specificity compared with healthy individuals. This finding provides practical guidance for sampling design in future acne microbiome studies, and suggests that the forehead and glabella can be prioritized as characteristic sampling sites. Targeted interventions based on area-specific microbial differences remain speculative at present and need to be verified in clinical trials. In future research, high-resolution techniques such as metagenomics and qPCR, combined with prospective longitudinal study designs, can be adopted to overcome the limitations of the current study and further elucidate the pathogenic mechanisms of acne.

## Data Availability

The raw sequence data reported in this paper have been deposited in the Genome Sequence Archive (The GSA Family in 2025: A Broadened Sharing Platform for Multi-Omics and Multimodal Data, 2025) in National Genomics Data Center (Database Resources of the National Genomics Data Center, 2026), China National Center for Bioinformation / Beijing Institute of Genomics, Chinese Academy of Sciences (GSA-Human: HRA019368) that are publicly accessible at https://ngdc.cncb.ac.cn/gsa-human.
